# Between a Rock and a Hard Place: COVID-19 Lessons Learned From Providers Rounding at Skilled Nursing Facilities in the Rural Midwest

**DOI:** 10.7759/cureus.32157

**Published:** 2022-12-03

**Authors:** Adria Whiting, Katie M Pace, Kimberly Edel, Madhan Prabhakaran, Marie L Steffl, Janice Shelton, Julie Pace, April E Poolman, Sheila E Anderson

**Affiliations:** 1 Family Medicine, Mayo Clinic Health System, Fairmont, USA; 2 Family Medicine, Mayo Clinic Health System, Mankato, USA; 3 Family Medicine, Mayo Clinic Health System, Belle Plaine, USA; 4 Family Medicine, Mayo Clinic Health System, St. James, USA; 5 Family Medicine, Mayo Clinic Health System, St. Peter, USA

**Keywords:** healthcare hero, infection control and prevention, mental health, healthcare workers, nursing homes, skilled nursing facilities, covid-19 retro

## Abstract

On March 11, 2020, coronavirus disease 2019 (COVID-19) was classified as a pandemic, setting in motion unprecedented practice changes across the healthcare industry. Never was this more evident than in Skilled Nursing Facilities (SNFs). SNFs were tested on multiple fronts, requiring innovation and perseverance at levels never before seen. Lessons learned from this setting to better prepare for the next pandemic include: updating and standardizing infection control and prevention policies, ensuring the supply chain keeps up with demand, updating infrastructure, creating a work environment that promotes well-being, and having clear communication plans.

## Editorial

Introduction

The coronavirus disease 2019 (COVID-19) pandemic has presented several challenges to the healthcare profession and changed the practices and processes related to healthcare delivery [1-4]. Some of the greatest challenges and lessons learned come from the population most greatly impacted by COVID-19, those living in skilled nursing facilities (SNF). This typically older and vulnerable population has been the most disproportionally affected by this pandemic, developing severe COVID-19 sequelae and having sustained some of the highest mortality rates worldwide [5,6]. SNFs have faced multiple unprecedented challenges, including shortages of critical supplies, quarantining large numbers of infectious residents, caring for those affected, and adhering to rapidly evolving recommendations while protecting residents and staff at unprecedented levels [7-9]. Measures to help mitigate the spread of the virus were rapidly deployed, including social distancing, requiring face masks, and quarantining the infected [3,4]. From the first wave of the pandemic through today, healthcare providers, SNF patients, and their families have had to develop solutions to address both unforeseen and planned challenges of the pandemic [10-15]. Almost immediately, frontline workers in hospitals, intensive care units, and emergency departments were hailed as heroes and their resilience recognized [9,16,17]. This article is designed to highlight six lessons learned from the COVID-19 pandemic by a division of providers and staff dedicated to patient care in SNFs located in rural Southwest Minnesota (SWMN).

1. Infrastructure Is Key to the Mitigation of Infection

Reflecting on COVID-19 in long-term care has revealed multiple gaps and areas that need improvement to better prepare SNF facilities for the next pandemic [7]. Gaps noted in SNFs serviced by our team include supply chain shortages, staffing shortages, poor bandwidth affecting telehealth, and outdated layouts of older buildings. Nationwide supply chains quickly collapsed amid the pandemic-driven demand for personal protective equipment (PPE) and medical supplies [7,9]. SNF staff reported to our rounding team the need to save masks for use due to PPE shortages. Several facilities our team provided rounding services to continued to have double occupancy rooms during the pandemic and were unable to support isolating infected individuals due to structural limitations of their facility's layout. To mitigate the spread of COVID-19, our team rapidly implemented the use of telemedicine in our facilities in March 2020. While telemedicine supplied a much-needed stopgap, it was not a perfect alternative. Telemedicine was found to be more favorable for some specialties than others, as it had limitations on the physical assessment of residents [1]. Additionally, we found that these rural facilities lacked the bandwidth to support telemedicine services. Short-staffed SNF nurses were relied on to provide those assessments and to facilitate the virtual visit [1]. This was a significant oversight when one of the steps to mitigate the spread of COVID-19 in the community was to keep COVID-19-positive residents in the SNF. These changes decreased the burden of the hospital system but at the same time, placed an even heavier burden on the SNF staff. This pushed an already chronically understaffed industry to near critical levels and ultimately the National Guard was deployed to help at some of our SWMN sites [3]. To build a more robust infrastructure, considerations should include strategies for procuring PPE and keeping supply chains open to rural areas, developing a more robust telemedicine platform, including improving rural internet services to maintain bandwidth, developing standardized infection control and isolation protocols that take into consideration older building layouts, optimizing standard staffing resources, and creating staffing protocols for acute care needs [2,6,7]. 

2. Communication Must Be Clear

The need for clear and concise communication became a top priority during this pandemic. The quality of the communication between our healthcare team (made up of the SNF staff and rounding providers) and the residents and their families varied between sites. Poor communication worsened pre-existing stress and worry among residents and family, further compounding the profound sense of isolation already felt [10]. Staffing at the sites our team serviced fluctuated based on COVID-19 outbreaks at each facility. This impacted the accuracy and timeliness of communication to rounding providers, residents, and their families. Our team recognized that SNF sites with improved communication included those with a consistent rounding nurse and a consistent rounding provider versus a different nurse and rounding provider every day. Our team found direct communication via telephone with the provider was also key to timely notification of outbreaks and condition changes. This led to meeting the residents' needs at the time they needed it most. Our team had on-call advance practice providers that provided hourly coverage via telephone visits and telemedicine visits when accessible. When possible, family members were included in the telemedicine appointment or called directly by the provider. This direct communication to the family aided in timely updates and family inclusion in the plan of care. Our team met weekly to discuss wins and opportunities for improvement at each site. Lessons learned by our team regarding communication for future pandemics include consistent rounding staff, access to direct communication with medical rounding providers, direct access to a provider, and routine intentional team meetings.

3. Mental Health Preparedness for Health Care Workers (HCW) Cannot Be Understated

Naturally, healthcare workers like to know the answers. It has always been our job to find answers to a patient’s symptoms, diagnose, and treat [13]. The pandemic, however, was full of an overwhelming number of unknowns leading to anticipatory anxiety that negatively impacted the mental health of HCWs and increased stress levels [10]. The unknowns were many - what did it mean to become sick with COVID-19? What would this mean for our workload? How would we treat patients? Would we have enough supplies? Would we have enough staff? Would we bring it back to our families at home? Would we be able to care for our families? [10].

HCWs stretched past their limits have led to increased burnout, more mental health disorders, and psychological trauma [18]. Burnout is not a new or limited phenomenon to the pandemic but has been exacerbated by the numerous factors that contribute to the stress healthcare professionals are currently facing. The international classification of disease (ICD)-11 defines burnout as “a syndrome conceptualized as resulting from chronic workplace stress that has not been successfully managed” [18]. We have seen and heard the reports that HCWs were being stretched too thin, emotionally and physically exhausted, worried about exposing loved ones, and not getting enough emotional support. In the SNFs, we saw this at an alarmingly higher rate among staff due to fewer resources available on a national level [7-9]. This pushed several employees into early retirement, leaving for employment elsewhere, and even some to leave healthcare altogether. In the state of Minnesota, there was an initiative of over 400 National Guard members who were sent to long-term care facilities across the state to help with the staffing.

As the COVID-19 pandemic continued surge after surge, the cheers that were so loudly publicized in the beginning have long since faded. The coined term “healthcare hero” once meant to be a term of the highest praise, soon became a barrier for staff feeling like they could not seek help for mental health. Now realizing the impact of a pandemic on HCWs, efforts toward substantial and long-term strategies focusing on mental health need to be implemented [18]. In particular, efforts aimed at reducing burnout and promoting workforce well-being. Encouraging daily self-care, self-awareness, self-compassion, practicing altruism, and checking in on one another are a few strategies [18]. However, first, we must start with breaking the stigma and allowing a safe place for HCWs to be open and vulnerable about the difficulties of working in this industry.

4. Crisis Can Spur Innovation

When the World Health Organization characterized the COVID-19 outbreak as a pandemic on March 11, 2020, it unknowingly changed the course of the healthcare delivery system. At that time, it was recommended that SNF patients avoid leaving the facility to prevent contracting and transmitting COVID-19. This included canceling all specialty appointments and preventative medicine appointments that require the patient to leave the facility. Providers were also directed to try to treat skilled nursing facility patients in the facility, instead of transferring them to the emergency department. The Centers for Disease Control and Prevention (CDC) noted that by June 30, 2020, there was an estimated 41% of US adults who delayed or avoided medical care [19].

The Public Health Emergency Waiver permitted providers to perform medical visits virtually, allowing for continuity of care as well as fulfilling Center for Medicare and Medicaid Services (CMS) regulatory requirements. It also waived the three-day prior inpatient hospital stay to qualify for Medicare Part A [20]. Patients no longer had to be admitted to the hospital to qualify for a skilled stay in an SNF [20]. Within weeks, our team implemented telemedicine to continue to see patients in the skilled nursing facility setting. This allowed providers to continue to have visits with patients, and address acute and chronic medical conditions while eliminating face-to-face exposure and the potential of spreading the virus. One study found that long-term care facilities that used telemedicine and remote monitoring to treat residents reduced hospitalizations and mortality compared to long-term care facilities that did not employ telemedicine [21]. Additionally, regulations surrounding home health were eased, allowing for advanced practice providers (APP) to certify the eligibility for beneficiaries to receive home health [20]. The easing of regulations allowed for more nimble transitions of care from hospital to SNF and from SNF to home, allowing for the appropriate care in the appropriate environment in a more efficient manner. To help with future pandemics, evaluation of regulations that would impede similar transitions of care now and in the future require a closer look for updating and revision.

5. Each SNF Must Have an Infection Preventionist

Challenges with how to best prevent and manage infection control in the SNF setting are ones that predate COVID-19 [6,7]. Long-term care remains a very high human touch industry with a high care burden [6,7]. Factors that contribute to barriers to infection control observed by our team include the communal living environment, the large number of staff needed to care for the residents, including certified nursing assistants (CNA), licensed practical nurses (LPN), registered nurses (RN), dietary staff, therapy staff, activity staff, social work, environmental services, maintenance staff, and the number of volunteers and visitors entering the facility. This is similar to findings found in the literature [22]. Additional contributors are residents with multiple medical comorbidities that increased infection risk whether through immune suppression or cognitive impairment that leaves affected residents unable to comply with the infection control policies meant to protect them.

When COVID-19 entered the SNF, it was catastrophic. SNFs became the epicenter of the pandemic. COVID-19 exposed the precariousness of this situation to the public eye, forcing administrators to re-examine infection control practices. Pandemic-related issues specific to rural SNFs that compounded infection control practices include varied interpretations of rapidly changing guidelines, differing visitor restrictions, and varying infection control and isolation practices at each SNF site. From an infection prevention point of view, we need to start by identifying how strong our current infection prevention strategies are in the SNF setting and ensure there is an infection preventionist for every site [6,7]. It would be important to then leverage leadership to help perform additional needs assessments that include communication, equipment resources, staffing resources and training, and community outreach [22,23].

6. Resilience Needs Support to Continue

Resilience defines the essence of those healthcare workers who found themselves on the front line of the COVID-19 pandemic. At the beginning of the pandemic, healthcare workers in SNFs found themselves on the front line, attempting to protect and care for both themselves and their vulnerable residents from a deadly virus [6-8]. With minimal PPE, equipment, and staff, and near-daily communication of evolving recommendations from the latest research, SNF staff and our rounding providers needed to adapt quickly to the constant changes [10]. The Minnesota Department of Health provided weekly phone calls with updates on these recommendations along with a questions and answers session. Our team had weekly phone calls to also discuss changes, challenges, and successes specific to the COVID-19 pandemic. In an attempt to protect SNF residents, visitors' access was restricted. This led to isolation, feelings of disconnect, confusion, and frustration for both the patient and their loved ones. The activity staff at many SNFs serviced by our provider team became innovative in providing connections for residents. Several solutions included the implementation of window visits, outdoor visits, and virtual visits with family members. They facilitated a connection to the outside world by taking residents’ pictures with messages on whiteboards and posting them on social media allowing family members across the nation to see their loved ones. Activities were changed to one-on-one visits and hallway BINGO where residents would sit in their doorway and a BINGO caller would be at the end of the hall.

While SNF staff and clinicians have shown extraordinary resilience and innovation in response to COVID-19, this pandemic has highlighted the prevalent pervasive challenges HCWs face in this industry [6-8,10]. The acute and prolonged stress of working under such surge conditions has escalated chronic stress and “moral injury” already endemic among healthcare professionals, challenging the long-term well-being and stability of the healthcare workforce [18]. In the future, it is imperative that HCWs in the SNF have programs to support the emotional well-being of both themselves and their residents during times of increased stress [18]. This needs to begin with ensuring the availability of mental health specialists at facilities, either in person or virtually, to provide support [9]. Additionally, focusing on more diverse and inclusive activities that are individualized to the resident’s plan of care would help ameliorate the disconnect from their familiar routine.

Conclusion

Despite the toll the COVID-19 pandemic has taken at all healthcare levels, its impact on the long-term care industry cannot be overstated. It has changed the face of practice management and consultation strategies. Telemedicine and virtual visit care have expanded exponentially since the pandemic started, but it has also highlighted the inequitable access to internet platforms in rural areas. The pandemic has revealed many opportunities for improvement, especially in long-term care. The need for infrastructure development, expanding access through virtual care, supply chain management, staffing challenges, prioritization of mental health, infection control standardization, and emergency preparedness are some core areas that deserve radical improvements. The pandemic has made us look back at our values and the characteristics that define us.

COVID-19 has indeed revolutionized the practice of medicine and helped us learn and understand the inadequacies that needed new perspectives and adaptations. The lessons learned from this pandemic experience have changed the outlook of medicine for future healthcare workers and emphasized better communication and workflow strategies for enhanced interdependency at work, developing emotional intelligence, self-care, and social responsibilities, and boosting the sense of community. The COVID-19 pandemic has challenged the lives of many; nonetheless, the healthcare industry has put on a united front and is on an evolutionary path of innovation and excellence.
